# B cells modulate lung antiviral inflammatory responses via the neurotransmitter acetylcholine

**DOI:** 10.21203/rs.3.rs-4421566/v1

**Published:** 2024-06-25

**Authors:** Nicole Baumgarth, Antonio Cembellin Prieto, Zheng Luo, Heather Kulaga

**Affiliations:** Johns Hopkins University; Johns Hopkins University; University of California Davis; Johns Hopkins University

## Abstract

The rapid onset of innate immune defenses is critical for early control of viral replication in an infected host, yet it can also lead to irreversible tissue damage, especially in the respiratory tract. Intricate regulatory mechanisms must exist that modulate inflammation, while controlling the infection. Here, B cells expressing choline acetyl transferase (ChAT), an enzyme required for production of the metabolite and neurotransmitter acetylcholine (ACh) are identified as such regulators of the immediate early response to influenza A virus. Lung tissue ChAT + B cells are shown to interact with a7 nicotinic Ach receptor-expressing lung interstitial macrophages in mice within 24h of infection to control their production of TNFa, shifting the balance towards reduced inflammation at the cost of enhanced viral replication. Thus, innate-stimulated B cells are key participants of an immediate-early regulatory cascade that controls lung tissue damage after viral infection.

## Introduction

Respiratory tract infections with influenza A or COVID-19 can lead to severe respiratory illnesses due to dysregulated inflammatory responses, including overshooting production of proinflammatory cytokines such as TNFa (so-called “cytokine storm”) by cells of the innate immune system. Interstitial lung macrophages play a particular role as elaborators of pro-inflammatory cytokines, as targets of COVID-19 viral infection, and as cellular organizers of the local healing response^1,2^. On the other hand, TNFa production by macrophages has been associated also with increased viral control after influenza infection, highlighting the need to balance innate immune activation for viral clearance with that of control for overshooting inflammation^3–7^. Despite recent advances in understanding of inflammatory pathways, identifying control mechanisms of pulmonary inflammation, especially those associated with viral infections, remains an urgent need where poor prognoses are often associated with dysregulated inflammatory responses^8–13^.

B cells are known foremost for their antibody production^14–16^, a function critical for controlling respiratory tract pathogens. Yet they can act also as antigen-presenting cells and produce cytokines and metabolites. GM-CSF and IL10-production by B cells were shown to regulate early immune responses, including in the lung^17–24^. Production of metabolites and neurotransmitters g-aminotubiric acid (GABA) and acetylcholine (ACh)^25–29,30,31^ are additional but less well understood effector functions of B cells. ACh is of interest, as it is a neurotransmitter generated from choline and acetyl coenzyme A via action of choline acetyltransferase (ChAT)^32,33^ that functions as both, controller of autonomic body functions^34–38^ and as immunoregulator, functions that are increasingly explored therapeutically^39–43,44–53^. ChAT-GFP reporter mice demonstrated ChAT expression by a variety of leukocytes^31,54–60^ and T cell derived ACh was shown to regulate macrophage function in the spleen^55,56^. However, the most abundant ChAT expression among leukocyte in the steady state was seen among B cells^31^, yet their immune modulatory role is little explored. Here, ChAT-expressing B cells are identified as critical immediate early modulators of interstitial lung macrophages during influenza virus infection.

## TNFa regulates viral loads and is a target of ACh

During viral respiratory tract infections, interstitial (IMs) and alveolar macrophages (AMs) are critical cellular components of the innate line of immune defense^1^. Overshooting activation can cause a local and systemic cytokine storm with detrimental effects on host survival, while following insufficient activation of the innate response or infection with high viral loads may result in a failure to control virus replication, leading to enhanced CD8 T cell-mediated immunopathology potentially resulting in host death^61–68^.

**Figure 1:**
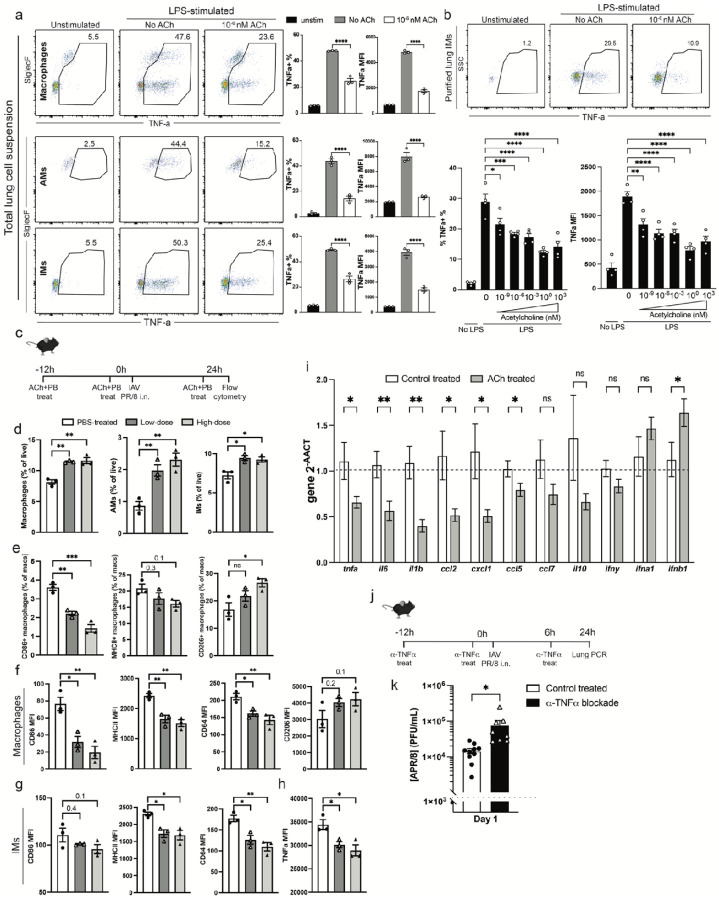
TNF-a regulates viral loads and is a target of ACh. a) total lung single cell suspensions from C57BL/6 mice (n=1–2) cultured in the presence or absence of LPS and treated or not with ACh, in the presence of Brefeldin A for 5h at 37°C. Representative flow cytometry plots (left) and frequencies of TNFa-expressing lung total macrophages (right, top; CD19−, ThY1.2−, Ly6G−, Ly6C−, F4/80+/CD64+), alveolar macrophages (AMs) (middle; further gated on CD11b−, CD11c+, SiglecF+), or interstitial macrophages (IMs; bottom; further gated on CD11b+, CD11c−, SiglecF−), plotted as frequency of the previous gate (left) or expressed as median fluorescence intensity (MFI, right). b) IMs from C57BL/6 mice (n=3–5) negativelyenriched with magnetic beads and cultured with indicated doses of ACh as in (a). Shown are representative flow cytometry plots (top) and frequencies of TNFa-expressing IM and TNFa MFI (bottom). c) Experimental design for (d-k). C57BL/6 mice were treated i. n. with a combination of ACh and Pyridostigmine Bromide 12h prior to, at the time of, and 24h after infection with 10PFU A/PR8 (0h), and lungs were analyzed via flow cytometry at 1 day post-infection. d) frequencies of lung parenchyma total macrophages, AMs and IMs (% of live) and e) frequencies of lung parenchyma macrophages expressing CD86, MHCII, and CD206, respectively. f) MFI of indicated markers in total macrophages and IMs, gated as in (a). h) MFI TNFa expression in IMs, ex-vivo re-stimulated with LPS in the presence of Brefeldin A for 4h at 37°C. i) relative gene expression of indicated genes in lung homogenates from Control-treated and ACh-treated mice at 1dpi with influenza A/PR8 n=6–8. j) experimental design for (k): C57BL/6 mice were treated with a blocking anti-TNFa antibody 12h prior to, at the time of, and 6h post infection with 10PFU APR/8. k) influenza A/PR8 viral loads (PFU/ml) as assessed by qRT-PCR on lung homogenates. a, b) representative of 3 independent experiments giving similar results, data contain n=3–4 total replicates per group. d-h) representative of 2 independent experiments with n=3–4 mice each. i-k) n=8–12 mice, pooled from 2 independent experiments k) c-j) Bar graphs show mean ± s.e.m. a-h) One-Way ANOVA. i-k) two-tailed unpaired Student’s t-test. *p<0.05; **p<0.01; ***p<0.001; ****p<0.0001; n.s. not significant.

To assess the effects of ACh on lung macrophage inflammatory responses, we applied ACh to either total lung leukocytes or enriched lung-derived IMs, which were also stimulated with LPS. Application of ACh to lung cell suspensions led to a more than 50% reduction in the frequencies of TNFa generating CD64 + F4/80 + total macrophages, as well as CD11b- CD11c + SiglecF + AMs and CD11b + CD11c- SiglecF- IMs (Fig. 1a). Consistent with a direct effect on macrophages, ACh reduced TNFa production by enriched lung IMs in a dose-dependent manner (Fig. 1b). Next, ACh and its stabilizer, the acetylcholinesterase (AChE) inhibitor pyridostigmine bromide (PB) were applied intranasally to mice 12h prior to, at the time of, and 24h after intranasal infection with influenza A/Puerto Rico/8/34 (A/PR8) (Fig. 1c). In this sublethal viral infection model, virus replication and innate-driven inflammation peaks within the first 2–3 days, and virus-dose dependent weight loss peaks around 7–9 days post-infection (dpi) (**Extended Data Fig. 1a**). Differences in macrophage responses following intranasal ACh application were observed consistently in AMs and IMs at 24h after infection in a ACh dose-dependent manner. Intranasally applied ACh affected macrophages in both, the airways and the lung parenchyma. Application of ACh enhanced AM numbers in the lung parenchyma (Fig. 1d), suggesting their migration from the airways into the tissue. Both, the frequencies (Fig. 1e) and expression levels (Fig. 1f) of costimulatory marker CD86 and of MHCII by total lung macrophages decreased in an ACh dose-dependent manner, while surface expression levels of the activation inhibitor CD206 + increased (Fig. 1e). IMs showed dose-dependent decreases in CD86, MHCII, and CD64 expression (Fig. 1g). Moreover, in vitro LPS stimulation of total lung single cell suspensions from ACh-treated mice showed decreased macrophage TNFa expression levels in an ACh dose-dependent manner (Fig. 1h). Consistent with decreased inflammation, qRT-PCR analysis of lung homogenates from ACh-treated mice showed significant decreased expression of pro-inflammatory genes *tnfa, il6, il1b*, and chemokines *ccl2, cxcl1, ccl5* and ccl7 compared to controls, while *ll10, ifng, ifna1* and *ifnb* were unaffected (Fig. 1i). Thus, ACh can modulate lung tissue AMs and IMs, reducing their levels of activation, as well as chemokine and cytokine expression within 24h of a respiratory tract viral infection.

TNFa and Nfkb signaling pathways are known regulators of early innate responses to influenza infection^69^. Consistent with those findings, lung IMs but interestingly not AMs, isolated from A/PR8 infected C57BL/6 mice at 1 dpi showed increased TNFa production after in vitro restimulation compared to those from non-infected mice. Increasing the dose of influenza A/PR8 used for infection also increased TNFa expression by IMs but again not AMs (**Extended Data Fig. 1b**). Early elaborated TNFa significantly contributed to control of influenza A/PR8 viral replication, as *in vivo* blockade with anti-TNFa mAb administered prior to and at the time of infection (Fig. 1j) significantly increased lung viral loads at 1 dpi compared to infected and sham treated C57BL/6 controls (Fig. 1k).

## B cells are the dominant ChAT expressing leukocyte population in the lung controlling Influenza A virus infection and TNFa production by interstitial macrophages

**Figure 2:**
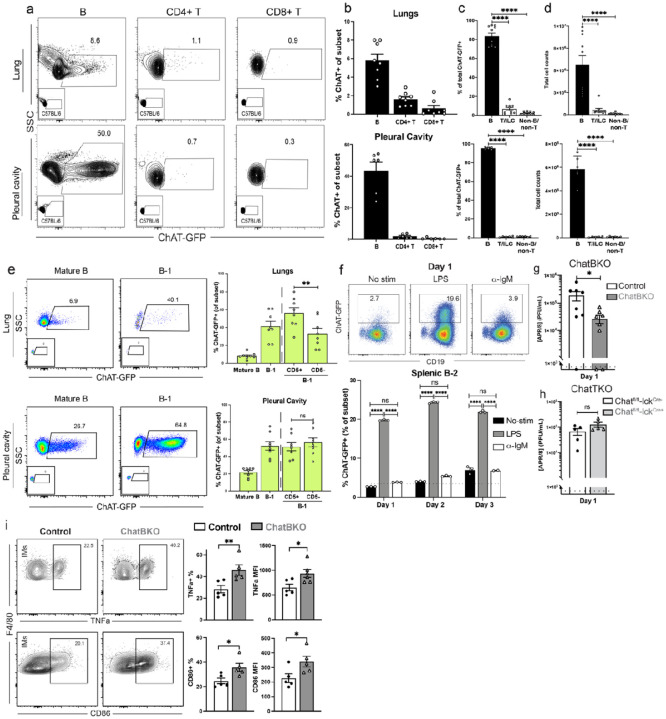
B cells are the major ChAT-expressing leukocytes and regulate Influenza A virus and interstitial macrophage TNFa. a) representative flow cytometry plots for the identification of ChAT-GFP+ B, CD4+ and CD8+ T cells from lungs (top) and pleural cavity (bottom) of ChATGFP reporter mice. Small inserts show same populations from non-GFP expressing C57BL/6 mice. b) summary of ChAT-GFP+ frequencies among CD19+ B cells, CD3+ CD4+ and CD3+ CD8+ T cells in lungs (top) and pleural cavity (bottom). b) frequencies of ChAT-GFP+ cells that are either B (CD19+), T/ILC (CD3+ or CD90.2+) or Non-B/Non-T (CD19− CD3−, CD90.2−). c) frequencies and d) cell counts of ChAT-GFP+ B, T/ILC and Non-B/Non-T cells in the lungs and pleural cavity. e) representative flow plots (left) and frequencies (right) of ChAT-GFP+ B cell subsets in the lungs (top) and pleural cavity (bottom). f) representative flow cytometry plots (top) and frequencies (bottom) of ChAT-GFP expression among magnetically enriched total splenic B- 2 cells (CD19+, CD23+, CD43−, CD5−, CD9−) from ChAT-GFP reporter mice cultured in the presence or absence of LPS, anti-IgM (Fab)2 or both, for indicated times. g-h) adult male and female mice were infected with 10PFU APR/8 intranasally (i.n.) and Influenza A/PR8 viral loads were analyzed by qRT-PCR on lung homogenates at 1dpi from (g) mb-1Cre−/− ChATfl/fl (Control) and mb-1Cre+/− ChATfl/fl mice (ChatBKO) (n=7–9) and (h) Chatfl/fl-lckCre−/− (Control) and Chatfl/fllckCre+/− (ChatTKO) mice (n=5). i) representative flow plots of lung tissue homogenates (left) and frequencies of TNFa-expressing lung IMs (right, top) or CD86-expressing IMs (bottom), after ex-vivo LPS re-stimulated in the presence of Brefeldin A for 4h at 37°C comparing mb- 1Cre−/− ChATfl/fl (Control) and mb-1Cre+/− ChATfl/fl mice (ChatBKO). a-e) n=6–10 mice pooled from 3 independent experiments. f) data contain n=3 replicates per group. g) n=7–9 mice, pooled from 2 independent experiments and h, i) n=4–6 mice pooled from 2 independent experiments. a-e) Bar graphs show mean ± s.e.m. except f. b-d, f) One-way ANOVA and e, g-i) two-tailed unpaired Student’s t-test. *p<0.05; **p<0.01; ***p<0.001; ****p<0.0001; n.s. not significant.

Given previous findings that T cell-derived ACh can modulate macrophage responses, we performed flow cytometry on pleural cavity lavage fluid, lung parenchyma, mediastinal lymph nodes (MedLN) and spleen of ChAT-GFP reporter mice^31^ to assess for the presence of leukocytes capable of generating ACh in the respiratory tract (Fig. 2a–d and **Extended Data Fig. 2a-d**). In all tissues analyzed, by frequency and total cell count, most ChAT + cells were CD19 + B cells (Fig. 2c, d and **Extended Data Fig. 2c, d**). In pleural cavity and lung parenchyma, nearly 50% and 10% of B cells, respectively, expressed ChAT (Fig. 2b). Unsupervised clustering of flow cytometry data revealed distinct clusters of ChAT-expressing B cells in the lung and pleural cavity (**Extended Data Fig. 2e-g**). ChAT + B cells were predominantly CD5+/−, CD19+, CD43+, IgM^hi^ and IgD^lo^, CD138-, thus mostly, albeit not exclusively, B-1 cells (Fig. 2e and **Extended Data Fig. 2h-l**), consistent with a previous study^31^. While B-1 cells dominated ChAT expression in the pleural cavity by frequency and total numbers, due to their larger total cell numbers, conventional mature B cells outnumbered B-1 cells in spleen, lung, and MedLN (**Extended Data Fig. 2h-l**).

In the bone marrow (BM), ChAT expression was observed only rarely among pro- and pre-B cells (Hardy fractions A-D) but was seen at increased frequencies at the immature B cell stage (B220^hi^ IgM+ IgD^−/lo^ CD93+; Hardy Fraction E) (**Extended Data Fig. 3a, b**). Similar to peripheral tissues, BM cells with a mature, B-1-like cell phenotype (CD45R+/lo CD93−, CD43+/−, IgDlo, IgM+) displayed the highest frequencies of ChAT expression in the BM (**Extended Data Fig. 3a, b**).

ChAT-GFP was induced in spleen FO B cells with LPS but only marginally after anti-IgM stimulation, consistent with previous data suggesting ChAT-expression was MyD88-dependent^31^ (Fig. 2f and **Extended Data Fig. 4a**). This may explain the relative large frequencies of ChAT-GFP + B-1 cells, as they respond more strongly to TLR-mediated signaling compared to conventional B cells and require MyD88 expression for survival and differentiation^70^. Stimulation of splenic B cells under conditions driving B cell differentiation reduced ChAT-GFP expression. This was seen following stimulation with both, LPS plus IL-4 and IL-5, as well as stimulation with anti-IgM, CD40L, IL4 and IL5 (**Extended Data Fig. 4b, c**). Similar results were obtained with sorted ChAT^neg^ B-1 B cells, ruling out preferential activation of ChAT negative B cells (**Extended Data Fig. 4d**). Consistent with these findings, plasmablasts and plasma cells obtained from respiratory tract draining MedLN of ChAT-GFP mice infected for 7 days with influenza A/PR8 lacked ChAT-GFP expression (**Extended Data Fig. 4e**). The data suggest an innate-like, immediate-early role for ChAT-expressing B cells, independent of further differentiation.

To determine whether B cell derived ACh regulates early respiratory tract responses to viral infections, we A/PR8 infected mice with a B cell-specific deletion of ACh (mb-1Cre^+/−^ ChAT^fl/fl^ mice, ChatBKO). ChatBKO mice showed 10-fold reductions in lung viral loads at 1 dpi with influenza A/PR8 compared to mb-1Cre^−/−^ ChAT^fl/fl^ control mice (Control, Fig. 2g), consistent with relatively high constitutive expression of ChAT by B cells prior to infection. While leukocyte-derived ACh effects in the spleen have been shown previously to require T cell-mediated ACh production^55,56^, T cell-specific deletion of ChAT (Chat^fl/fl^- Lck^Cre+/−^) did not affect influenza virus loads at 1 dpi (Fig. 2h), consistent with their low frequencies in all tissues prior to infection (**Extended Data Fig. 2**).

B cells remained the predominant population of ChAT-GFP + cells at early infection timepoints (**Extended Data Fig. 5a-e**). In addition, significant increases in absolute, but not relative, numbers of ChAT-GFP + B cells were measured at 1 and 3 dpi in the lungs of ChAT-reporter mice (**Extended Data Fig. 5a-c**). No accumulation of ChAT-GFP + T cells in the lungs were observed until 7 dpi, a time when influenza-specific CD4 T cells are known to enter the lung in large numbers and virus is largely cleared from the lungs (**Extended Data Fig. 5d, e**). Together these data indicate that innate signal-induced ChAT expressing respiratory tract B cells rapidly respond to innate stimulation to affect influenza A virus replication in the lung. If primed and activated ChAT + T cells affect immune responses to influenza infection via ACh release, they would not do so until later timepoints^71,58^.

Given the decreased control of influenza virus replication in mice in which TNFa signaling was blocked (Fig. 1j), the effects of B cells on control of macrophage function during influenza infection were evaluated by measuring TNFa production in μMT^−/−^ mice, which lack mature B cells in all tissues, including the lungs (**Extended Data Fig. 6a**). Cells isolated from the lungs of control and μMT−/− mice at 1 dpi with A/PR8 showed significant increases in TNFa production by IMs but not AMs from μMT mice following in vitro restimulation compared to controls, supporting a modulating effect of B cells on IMs but not AMs immediately early after infection (**Extended Data Fig. 6b**). μMT^−/−^ mice also showed increased lung monocyte infiltration and decreased AM numbers, suggesting that the absence of B cells caused enhanced inflammation and perhaps increased AM apoptosis (**Extended Data Fig. 6c**)^72–74^. Furthermore, μMT^−/−^ mice had reduced frequencies of macrophages expressing the inhibitory receptor CD206 and decreased CD206 expression levels, but higher surface expression of activation markers F4/80, CD11b and CD64 (**Extended Data Fig. 6d**) and in IMs (**Extended Data Fig. 6e**). Thus, B cells regulate the activation state of IMs, but not AMs, in the respiratory tract immediately early after influenza virus infection.

To determine the extent to which these effects of B cells were facilitated by their secretion of ACh, A/PR8 infected ChATBKO mice were analyzed at 1 dpi. Consistent with a major role for B cell derived ACh, ChatBKO derived IMs showed increased TNFa production as well as increased expression of CD86 very similar to IMs in μMT^−/−^ mice, while AMs were unaffected (Fig. 2i, **Extended Data Fig. 6f**). Moreover, qRT-PCR analysis of lung homogenates from ChatBKO revealed increased expression of pro- inflammatory cytokine genes like il6, tnfa, il1b, and csf2, while il10 showed minimal change or downregulation compared to controls. ifna1 expression was reduced in the absence of B cell derived ACh, consistent with reductions in lung viral loads in the ChatBKO mice compared to controls (**Extended Data Fig. 6g** and Fig. 2g).

Depletion of ChAT in B cells did not significantly affect neutrophil numbers at 1dpi with A/PR8 (**Extended Data Fig. 6h**), a population that was shown previously to be affected by B cell derived ACh in a sepsis model^31^. Slight increases in lung monocyte and neutrophil cell counts, however, were noted by 7 dpi in ChatBKO mice (**Extended Data Fig. 7a**). Histolopathological evaluation of the respiratory tract at 7 dpi suggested enhanced epithelial degeneration in the nasal cavities and poorer overall health of ChATBKO mice compared to the controls (**Extended Data Fig. 7b, c**). The analysis also showed heightened CD8 T cell infiltration into the lungs and increased NK cells in the spleen, indicating enhanced systemic inflammation (**Extended Data Fig. 7d, e**). This was despite similar lung viral loads of control and ChatBKO mice by 7 dpi (**Extended Data Fig. 7f**), suggesting insufficient control of lung inflammation as main causes of increased pathology and immune cell activation. Altogether, the data demonstrate that B cell derived ACh acts on IMs but not AMs to inhibit lung inflammatory responses to influenza infection at 1 dpi, inhibiting early control of lung viral loads, but significantly modulating production of various pro-inflammatory cytokines and chemokines and reducing the impact of the respiratory tract infection both locally and systemically.

## B cells regulate innate immune cells in the lung parenchyma via ACh production

Previous studies demonstrated that immediate early influenza virus infection is controlled in part by natural IgM production, secreted mostly by B-1 cells^75,76^, which we show here to be a ready source for ACh. However, lack of ChAT expression by B cells did not significantly affect total or virus-binding IgM levels (**Extended Data Fig. 8a, b**), further supporting an antibody independent role for B cells in inhibiting IMs’ ability to secrete TNFa and in promoting viral replication. Similarly, the lack of B cell-expressed ChAT had no effect on extrafollicular plasmablast development or the frequencies of germinal center B cells (**Extended Data Fig. 8c**), influenza-specific IgM or IgG antibody-secreting cells in the MedLN at 7 and 14 dpi (**Extended Data Fig. 8d**), nor on serum influenza-specific IgM or IgG subclasses over a 4-week time-course (**Extended Data Fig. 8e**). Deletion of ChAT in B cells also did not affect total numbers of innate leukocytes, T or B cell subsets in the spleen, or bone marrow B cell development when compared to control mice^77^ (**Extended Data Fig. 9a-e**). The exception was a slight but significant reduction in the number of CD5 + B-1 cells in ChATBKO mice compared to ChATflx/flx Cre-negative controls (**Extended Data Fig. 9f**). Studies with non-floxed and Cre-expressing mb-1^Cre−/−^ ChAT^+/+^ mice showed similar reductions, suggesting that this effect on B-1 cell numbers was driven by mb-1 haploinsufficiency rather than ChAT expression (**Extended Data Fig. 9g**)^78–80,81^.

**Figure 3:**
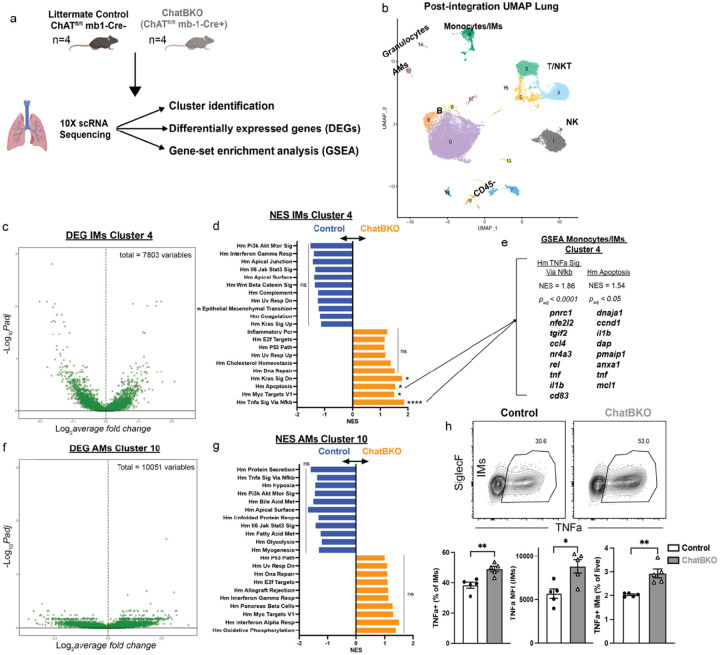
B cell derived ACh alters the transcriptional profile of innate lung immune cells. a) experimental approach b-g: live, single cell suspensions of lungs from mb-1Cre−/− ChATfl/fl (Control) and mb-1Cre+/− ChATfl/fl (ChatBKO) mice (n=4 mice/group adult females) were analyzed by single-cell RNA sequencing (scRNA-Seq). b) scRNA-Seq post sample-integration uMAP plots of all lung parenchyma cells. c) differential gene expression among cells in cluster 4 (IMs) and (f) 10 (AMs), comparing Control and ChatBKO mice. d, g) 10–11 selected most differentially expressed hallmark signaling pathways in (d) clusters 4 and (g) 10, comparing Control (blue, left) and ChatBKO (orange, right) mice as identified by gene set enrichment analysis (GSEA). e) Statistical analysis involving the calculation of padj-values and the determination of the normalized enrichment score (NES) for the two pathways exhibiting the most differential expression; 8–10 genes with the most differential expression between groups within each specified pathway h) representative flow cytometry plots (top) and quantification (bottom) of the steady state frequencies of TNFa-expressing lung interstitial macrophages (IMs) (CD19−, CD5−, CD11b+, F4/80+/CD64+ CD11c−, Ly6G−, Ly6C−, SiglecF−) plotted as a frequency of the previous gate (bottom left), MFI quantification (bottom, middle) and as a frequency of live cells (bottom right) ex-vivo LPS re-stimulated in the presence of Brefeldin A for 4h at 37C comparing Control and ChatBKO mice d-g) (refer to methods) fgsea v1.24.0 was used to run GSEA with gene sets obtained from the Molecular Signatures Database. Features were ranked by -log(pvalue) * sign(foldchange). h) n=5 pooled mice from 2 independent experiments. NES = normalized enrichment score. Bar graphs show mean ± s.e.m. h) two-tailed unpaired Student’s t-test *p<0.05; **p<0.01; ***p<0.001; ****p<0.0001; n.s. not significant.

To assess the impact of B cell-specific ACh generation on respiratory tract leukocytes we conducted single-cell RNA sequencing (scRNA-Seq), comparing cells from lung parenchyma of Control and ChatBKO mice prior infection (n = 4/group) (Fig. 3a). Post sample integration analysis revealed 16 distinct cell clusters within the lung parenchyma (Fig. 3b). Clusters 7, 9, 11, and 13 were CD45^neg^ and classified as non-immune cells, and the remainder were CD45 + leukocytes (**Extended Data Fig. 10a and Supplemental Data 1**). Among CD45 + leukocytes, B cells were identified as clusters 0, 6, 8, and 12, while T/NKT cells were present in clusters 15, 5, 2, and 3 and NK cells in cluster 1 (**Extended Data Fig. 10b-d and Supplemental Data 1**). Clusters 10, 14, and 4 represented myeloid cell compartments (**Extended Data Fig. 10e-h and Supplemental Data 1**). Cluster 10 exhibited markers indicative of AMs, cluster 14 granulocytes, and cluster 4 monocyte/monocyte-derived macrophages or IMs (**Extended Data Fig. 10e-h**). The cluster 4 IMs expressed markers of activation, including tnf, socs3, cd80, and cd86 (**Supplemental data 1**).

No notable differences were observed in lung cell subset frequencies between Control and ChatBKO mice (**Extended Data Fig. 10i**). To identify potential targets of B cell derived ACh, we compared the transcriptional profiles of lung parenchyma cell clusters from Control and ChatBKO mice. Amongst CD45^neg^ clusters, only cluster 11 showed significant differences in gene expression, as assessed by GSEA. Those differences included pathways associated with an IFN-g responses, IFN-a response and the PI3K-AKT- mTOR signaling pathway, all of which showed increases in the absence of B cell derived ACh (**Supplemental data 2**). Amongst the CD45^pos^ clusters, most B cells (cluster 0, 6 and 8) and CD8 T cells lacked significant changes (**Supplemental data 2**). NK and NKT cells (clusters 1 and 3) showed differential expression of immune-related pathways, including lower TNFa signaling via Nfkb pathway (**Supplemental data 2**). Amongst the myeloid clusters, AMs (cluster 10) showed no differences, while both granulocytes (cluster 14) and the biggest myeloid cluster (cluster 4), consisting of monocytes and IMs, showed the strongest differences between Control and ChatBKO mice with statistically significant differences in expression levels of 700 genes (Fig. 3c, **Extended Data Fig. 10j and Supplemental Data 2**). GSEA demonstrated upregulation of several hallmark pathways in IMs of ChatBKO mice, including kras signaling, myc targets V1, and notably, the apoptosis and the tnfa signaling via nfkb pathways (p_ad_j<0.05 and p_ad_j<0.0001) (Fig. 3d, e). The apoptosis genes dap, pmaip1, anxa1, mcl1, and activation and immune-related genes pnrc1, nfe2l2, ccl4, rel, tnf, il1b, and cd83 were genes driving the difference, consistent with the flow cytometric data, suggesting that inhibition of viral replication via TNFa may occur through increased apoptosis^82,83^. Also consistent with the functional data obtained after influenza infection, transcriptional analysis revealed no significant differences in the subset classified as AMs (Fig. 3f, g and **Extended Data Fig. 10k**). Thus, the lack of B cell derived ACh affected IMs but not AMs, further demonstrating the specific impact of ChAT + B cells on monocyte/monocyte-derived macrophages/IMs.

Flow cytometry supported the gene expression differences, with IMs from non-infected ChatBKO mice displaying increased TNFa production upon short-term restimulation with LPS in vitro, both by frequency and MFI, compared to controls (Fig. 3h and **Extended Data Fig. 11a-c**). Notably, the scRNA-sequencing data confirmed that changes among lung macrophages were restricted to IMs, as no significant differences were observed in AMs from ChatBKO and control mice regarding frequencies or total cells of TNFa producers, albeit a slight difference in TNFa MFI was observed (Fig. 3h and **Extended Data Fig. 11a-c**). Further characterization revealed significant increases in F4/80 expression on IMs and subtle differences in surface expression of CD11b and CD206 (**Extended Data Fig. 11b, c**) in cells from ChatBKO mice. These findings indicate that B cell derived ACh significantly alters the functionality of lung IMs, but not AMs, revealing a distinct regulatory pathway by which B cells regulate inflammatory responses in the respiratory tract.

## B cell derived ACh directly inhibits IMs via the α7 nicotinic ACh receptor (a7nAchR)

Given the overall ability of AMs to respond to ACh (Fig. 1a), the data suggest that the distinct and selective effects of ChAT + B cells on some myeloid cell clusters was driven by the location of B cells in the respiratory tract, rather than their differential ability to respond to ACh. Indeed, B cells are readily found in the lung interstitium, in fact they constituted the largest cluster of leukocytes by scRNAseq (Fig. 3b), but they are not present in the airways of not previously infected mice^84,85^. Given the extremely short half-life of ACh, this suggests that ChAT + B cells may exert a direct effect on IMs.

**Figure 4:**
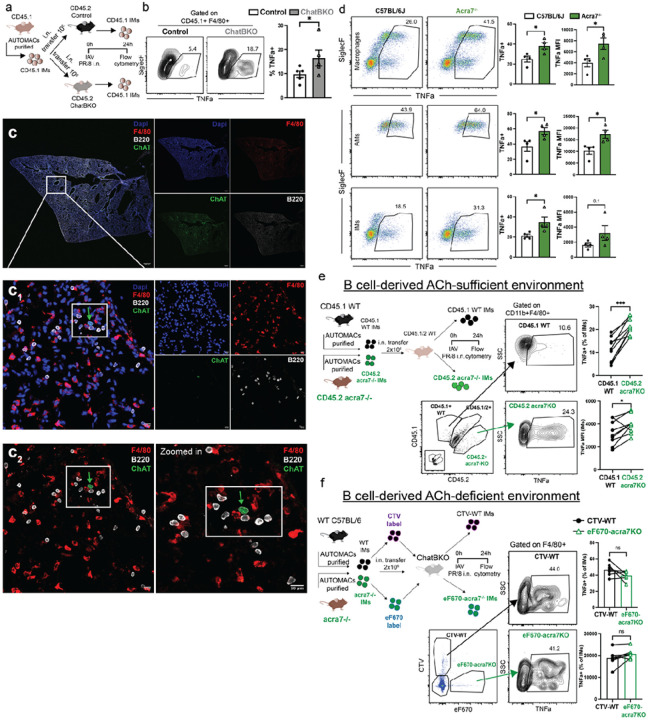
B cells directly interact with lung IMs via ACh and a7nAChR during early influenza infection to regulate inflammatory responses a) Experimental design for b). b) representative flow cytometry plots (left) and summary of frequencies (right) of CD45.1 TNFaproducing lung interstitial macrophages (IMs) (CD45.1+, CD45.2−, F4/80+) adoptively transferred into CD45.2+ WT or ChatBKO mice at 1dpi with influenza A/PR8 after restimulation of lung tissue with LPS in the presence of Brefeldin A for 4h at 37C. c) Shown are representative maximum projection lung immunfluorescent (IF) images (10x) of ChAT-GFP reporter mice at 1 dpi with influenza A/PR8. Blue (DAPI), Red (F4/80), White (B220) Green (ChAT-GFP). Scale bar 500μm. Right panels represent single channels as indicated. Boxes indicate area of focus. c1) representative 63x oil z-stack maximum projection image, scale bar 10μm. Right panels represent single channels as indicated. Boxes indicate area of focus and arrows indicate ChATexpressing B cells (Green, ChAT-GFP) in vicinity with macrophages (Red, F4/80) c2) representative z-stack maximum projection lung IF images from b) zoomed in image (right). Scale bar 10μm. d) (left) representative flow cytometry plots and (right) summary of frequences and MFI (right and far right) of TNFa expression by (top) macrophages (CD19−, Thy1.2−, Ly6G−, Ly6C−, F4/80+/CD64+), middle AMs (further gated on CD11b−,CD11c+, SiglecF+), or bottom IMs (further gated on CD11b+, CD11c−, SiglecF−) from lungs of 24h A/PR8 infected WT C57BL/6 and acra7−/− mice (n=4) restimulated in vitro for 4h with LPS in the presence of Brefeldin A. e) left, experimental design; right and bottom, representative flow plots from lung macrophages after gating for CD45 allotype expression to identify cellular origins from either WT (CD45.1), acra−/− mice (CD45.2) or host (CD45.1/2) and staining for TNFa after in vitro restimulation as for d). Far right, summary of frequencies TNFa (top) and MFI (bottom), pairing results of adoptively transferred cells into the same recipient. f) left, experimental design; middle, representative flow plots of gating strategy identifying differentially dye-labeled WT C57BL/6 (CTV) and acra7−/− (eF670) lung IMs 24h after adoptive transfer into influenza A/PR8 infected ChatBKO mice. right, summary of frequencies TNFa+ lung IMs after ex-vivo LPS re-stimulation as for , e). a, b, d) representative of 2 independent experiments with similar results n=4 each. c) representative of 3 independent experiments with similar results. e, f) n=6–8 mice pooled from 2 independent experiments. Bar graphs show mean ± s.e.m. a, b) One-tailed unpaired Student’s t-test d) twotailed unpaired Student’s t-test e, f) two-tail paired Student’s t-test *p<0.05; **p<0.01; ***p<0.001; ****p<0.0001; n.s. not significant.

To assess this, allotypically marked lung IMs from CD45.1 + C57BL/6 wildtype (WT) mice were enriched to > 75% by magnetic cell separation and adoptively transferred intranasally into either CD45.2 + C57BL/6 WT controls or CD45.2 + ChatBKO, followed by infection with influenza A/PR8. 24h later, lung single cell suspensions were stimulated in vitro for 4h in the presence of LPS and Brefeldin A to assess cytokine production by the adoptively transferred macrophages (Fig. 4a). Significantly greater TNFa responses were seen from the transferred IMs placed into ChatBKO compared to those placed into Control mice (Fig. 4b). Thus, ruling out differences in IMs development and/or epigenetic changes in ChatBKO mice as a reason for their enhanced inflammatory responses following influenza infection. In further support of direct B cell – IMs interaction, confocal microscopy revealed the co-localization of B220 (CD45R) + B cells and F4/80 + macrophages in the lung interstitium of ChAT-GFP reporter mice at 1 dpi with influenza (Fig. 4c and **Supplemental Fig. 3**). GFP + B cells were often seen among small clusters of GFP- B cells, and in close proximity to one or more F4/80 + macrophages (Fig. 4c and **Supplemental Fig. 3**). These small clusters do not represent bronchus associated lymphoid tissues, which are not typically observed this early after infection of naïve mice.

Cells respond to ACh via several nicotinic and/or muscarinic cholinergic receptors. The inhibitory effects of T cell derived ACh on splenic macrophages were shown to depend on the a7 nicotinic (n)ACh receptor (R)^86–92^. Revealing a role for a7nAChR also in controlling inflammatory responses of lung macrophages by ACh, increased TNFa generation was observed in both AMs and IMs from 1 day influenza A/PR8 infected mice that lacked this receptor (acra7−/−) compared to controls (Fig. 4d).

Additional IM cell adoptive transfer experiments were conducted to probe further for a direct impact of B cell derived ACh on IMs (Fig. 4e–f). For that equal numbers enriched IMs from allotype disparate 45.2 + acra7−/− mice and CD45.1 + WT controls were transferred i.n. into WT CD45.1/2 double-positive C57BL/6 mice which were then infected with influenza A/PR8 (Fig. 4e). 24h later, lung cells were stimulated in vitro with LPS, demonstrating significantly enhanced TNFa production in the CD45.2 + acra7−/− IMs compared to the co-transferred WT CD45.1 + cells (Fig. 4e). In contrast, when cell tracker dye labeled WT (CTV) and α7nAChR-deficient (eF670) CD45.2 + IMs were co-transferred into ChatBKO mice, which were subsequently infected for 24h, no significant difference in TNFa production was seen between acra7−/− and WT cells (Fig. 4f). We conclude that B cells directly modulate cytokine production by lung IMs via ACh production immediately early after a respiratory tract infection.

## Discussion

Immediate early induction of cytokines and chemokines is needed to orchestrate a strong and protective innate immune response to viral infections. The quality and magnitude of this response not only directly affects virus replication it also significantly effects the long-term consequences of this immune activation, balancing the need for viral replication control with the requirement to avoid host tissue damage. Lung IMs are increasingly recognized as critical components of infection-induced inflammatory cytokine and chemokine responses as well as facilitators of tissue repair. Their controlled activation is thus critical in balancing host immune responses. Here we demonstrate a new regulatory axis by which a7AChR + lung macrophages are regulated by ChAT + lung tissue B cells, the latter found co-localized with macrophages in the lung parenchyma but not the airways (**Extended Data Fig. 12**). The B cells’ ability to generate ACh appears to directly regulate IM activation and TNFa production, and with it both, the control of influenza virus replication. Its absence resulted in enhanced elaboration of other pro- inflammatory cytokines in the lung, as well as later in infection increased local T cell infiltration and respiratory tract pathology, as well as increased systemic NK cell activation.

The two most prominent signaling pathways of lung interstitial macrophages enhanced in ChATBKO mice were the apoptotic pathway and the TNFα signaling via NF-κB pathways. Treatments targeting TNFα and IFNγ have shown promise in reducing mortality by attenuating cytokine storms in both mice and humans^3–5,93–96^, although their precise mechanisms remain unclear^6,7,69,97–99^. Furthermore key clinical trials using corticosteroids in COVID-19 patients highlighted the critical need for balancing inflammation while controlling viral replication^61–68^. Our studies suggest that B cells can provide highly targeted control of lung IM function^100,101,102,10^.

The study adds to the growing list of immunomodulatory functions attributable to ACh; it also adds another antibody independent, immune regulatory function to B cells. ChAT expression by B cells in vitro was promoted by TLR4 stimulation in by both B-1 and B-2 cells, consistent with the dependency of B cell ChAT expression on MyD88 expression^31^. However, it remains to be determined whether TLR stimulation is the only innate signal responsible for ChAT expression by B cells. When B cells underwent activation and plasma cell differentiation in vitro following ligation with anti-BCR or IL-4, IL-5, and CD40L, TLR-induced ChAT expression was inhibited, suggesting that ChAT-induction is promoted only in B cells that do not participate in the T-dependent, antigen-specific B cell response. Thus, perhaps suggesting a division of labor, in which antigen-specific B cells remain ChAT negative and differentiate instead into antibody-producing plasmablasts and plasmacells, while those activated by innate signals, but not stimulation via the BCR, may be recruited into early innate immune response regulation. This would be consistent with the findings that many ChAT + cells belong to the B-1 cell subset, a cell population that does not respond to BCR signaling with clonal expansion^103^, and immature B cells, another cell population that does not respond to BCR signaling with activation and differentiation unless rescued by provision of T cell help or other costimulatory signals^104–106^. Intriguingly, IL-10 producing B cells (Bregs)^22,107,108^ are also most prevalent among B-1 and Immature B cells, and only rarely FO B cells. However, IL-10 production was found also among certain plasma cells^109–111^.

ChAT + B cells store ACh in cytoplasmic vesicles^77^ from which they can be released rapidly via stimulation with the intestinal neuropeptide cholecystokinin (CCK)^31^. What regulates ACh release by B cells in the lung parenchyma remains to be defined but may involve other neuropeptides, as B cells express receptors for neuropeptides that can be released by specialized sensory nociceptive neurons and pulmonary neuroendocrine cells in the respiratory tract^29,112^. Of note, we found differences between the transcriptional profile of various myeloid cell populations in the lungs of ChATBKO mice compared to controls already prior to the influenza virus infection, suggesting the continuous release of ACh from B cells at this site, perhaps triggered by continuous environmental stimuli.

Previous studies showed that ACh can inhibit the JAK2-STAT3 pathway, although this inhibition was not entirely dependent on SOCS3, which is known to dephosphorylate STAT3^92,113–115^. Another study identified ACh’s effect on the nAChR/pERK pathway and the promotion of IL-10 production^42^. However, ACh does not seem to affect IL-10 in splenic or cavity macrophages^90^. Similarly, we found no difference in IL-10 transcript levels in ACh-treated mice, suggesting that the nAChR/pERK pathway might be specific to myeloid-derived suppressor cells, and in this case might work through JAK2-STAT3, or another unidentified pathway. Future targeted analysis and experimental approaches are needed to investigate the specific mechanisms of ACh’s regulation of TNFa production and apoptosis pathways in IMs and how this contributes to overall inflammation and viral control during respiratory viral infections. Such advances could increase our understanding of lung inflammatory response regulation and identify potential new therapeutic targets and biomarkers of such diseases.

The known cholinergic anti-inflammatory pathway involves acetylcholine receptor (AChR) agonists inhibiting cytokine expression in human macrophages and in septic rats and mice, leading to improved survival^86,89–92,116,117^. Later studies emphasized the crucial role of the α7nAChR in inhibiting inflammation and preventing sepsis^87,88,118,119^. These effects have shown effectiveness in mitigating conditions such as rheumatoid arthritis, inflammatory bowel disease, and colitis, in both mice and humans^39–43^. Given the known role of ACh derived from T lymphocytes in regulating splenic macrophage TNFa production, the lack of effect of T cell derived ACh on macrophage function in the lung after influenza infection was somewhat surprising. However, this is consistent with the finding that most ChAT-expressing leukocytes are B cells in the respiratory tract, spleen, and MedLN both at steady state and at 24hours after influenza infection. Moreover, all ChAT-expressing T cells had an activated CD44hi CD62Llo phenotype, a cell population that does not appear in the lungs of infected mice until around day 5–7 of infection. Thus, indicating profound differences in the kinetics and regulation of ACh production by B and T cells.

In conclusion, our studies indicate that B cell derived ACh regulates IMs during respiratory tract viral infections, cells that are increasingly identified as critical sources of protective innate but also harmful inflammatory responses and misdirected tissue repair leading to increased lung fibrosis following infections with respiratory tract pathogens such as influenza virus and COVID-19. Despite the broad presence of ACh in neuronal synapses in all tissues, and strong expression of the numerous cholinergic receptors on many cell types, its regulatory effect on lung IMs during early infection depends on secretion by B cells that reside in close proximity in the same tissue space, revealing a new, highly cell and location-specific regulatory axis controlling lung inflammation.

## Methods

### Mice

Male and female 8–14 wk-old C57BL/6J (CD45.2 #000664), C57BL/6J-Ptprc^em6Lutzy^/J (JAXBoy CD45.1 #033076), B6.Cg-Tg(RP23–268L19-EGFP)2Mik/J (ChAT^BAC^eGFP #007902), B cell-deficient (uMT #002288), B6;129-Chat^tm1Jrs^/J (ChAT^flox^ #016920), B6-C(Cg)-Cd79a^tm1(cre)Reth^/EhobJ (Mb1-Cre on C57BL/6 #020505), B6.Cg-Tg(Lck-cre)548Jxm/J (Lck-Cre 548-O #003802) mice were commercially obtained from The Jackson Laboratories.

Strains B6;129-Chat^tm1Jrs^/J (ChAT^flox^ #016920), B6-C(Cg)-Cd79a^tm1(cre)Reth^/EhobJ (Mb1-Cre on C57BL/6 #020505), B6.Cg-Tg(Lck-cre)548Jxm/J (Lck-Cre 548-O #003802) were initially provided by Drs. Colin Reardon and Kathrin Murray (UC Davis) and then continued to be bred in the animal facilities at Johns Hopkins University.

B6;129-Chat^tm1Jrs^/J (ChAT^flox^ #016920) were bred with B6-C(Cg)-Cd79a^tm1(cre)Reth^/EhobJ (Mb1-Cre on C57BL/6 #020505) to generate a B cell-specific deletion of *chat* and with B6.Cg-Tg(Lck-cre)548Jxm/J (Lck-Cre 548-O #003802) to generate a T cell-specific deletion of *chat*. C57BL/6J (CD45.2 #000664), C57BL/6J-Ptprc^em6Lutzy^/J (JAXBoy CD45.1 #033076) were bred to generate a CD45.1/2 strain for adoptive transfer experiments.

All mice were housed in SPF conditions in ventilated filtertop cages with food and water ad libitum at the University of California, Davis and the Johns Hopkins Bloomberg School of Public Health. Euthanasia was done by overexposing mice to CO_2_. All studies involving mice were conducted in compliance with, and after approval of protocols by the UC Davis Institutional Animal Care and Use Committee (IACUC) and by the Johns Hopkins University Animal Care and Use Committee (ACUC).

### Influenza infections

Mice were anesthetized and intranasally infected (i.n.) with influenza A/Puerto Rico/8/34 (A/PR8) virus. 10PFU/mouse were used, unless otherwise stated in the figure legend, in 40 μL PBS^120^ which was established as a sublethal dose generating on average less than 20% body weight loss over the course of infection.

### Tissue processing and flow cytometry staining

Lymph node and spleen cell suspensions were prepared as previously outlined^120^. Briefly, tissues were ground between the frosted parts of two microscope slides and then incubated in ACK lysis buffer for 1 minute on ice to eliminate erythrocytes. Subsequently, the cells were passed through a 50 μm nylon filter and diluted for staining.

For lung tissue collection, lungs were harvested after left ventricle perfusion of the heart and then mechanically and chemically digested. The lungs were placed in 3 mL of DMEM F12 1X with 10% NCS in gentleMACS^™^ M Tubes (Milenty, # 130-093-236) and processed using a gentleMACS dissociator m_Lung_02 twice (Milteny). Following this, the lungs were incubated with DNAse I (50 U/mL) (Worthington-Biochem # LS002139) and Collagenase, Type I (250 U/mL) (Worthington-Biochem # LS004196) for 25 minutes at 37°C at 220 rpm shaking. After incubation, the lungs underwent another round of processing using the m_lung_02 program. The resulting cells were passed through a 50 μm nylon filter and diluted for staining, similar to lymph node and spleen preparations.

Single-cell suspensions were incubated with Fc receptor block (anti-CD16/32, made in-house) and Live/Dead Fixable Aqua or Near IR (Thermo Fisher, L34967 or L34994) in PBS for 20 minutes on ice. Subsequently, the cells were stained with fluorescently labeled anti-surface receptor antibodies according to the manufacturer’s instructions regarding temperature and duration (see methods **Table 1** for the list of antibodies used) in staining media^120^. All fluorophore-conjugated antibodies were titrated before use to ensure maximal differential staining between the negative and positive fractions. For intracellular staining, the eBioscience^™^ Foxp3 Transcription Factor Staining Buffer Set (Thermo Fisher REF 00-5523-00) was used following the manufacturer’s instructions.

### Magnetic cell enrichment (auto-MACS) and ex-vivo cell cultures

Single-cell suspensions from the lung or spleen were prepared as described above and treated with anti-mouse CD16/32 for 20 minutes on ice to block Fc receptors. Subsequently, the cells were stained with biotinylated anti-surface receptor antibodies according to the manufacturer’s instructions regarding temperature and duration, in staining media. Anti-biotin microbeads were then added to the cells according to the manufacturer’s protocol, and the cells were passed through a 50 μm nylon filter before separation using the auto-MACS Pro Separator with the “depletes” option (Miltenyi Biotec # 130-092-545). Interstitial macrophages (IMs) from the lung were purified using anti-biotin magnetic beads after staining cells with biotinylated anti-CD90.2 (30-H12), -CD4 (GK1.5), -CD5 (53 – 7.3), -CD8a (53 – 6.7), -Gr-1 (RB6–8C5), -NK1.1 (PK136), CD49b (DX5), resulting in 70–75% purities as determined by subsequent flow cytometry analysis. Splenic B-2 cells were purified using biotinylated anti-CD90.2 (30-H12), -CD4 (GK1.5), -CD8a (53 – 6.7), -Gr-1 (RB6–8C5), -NK1.1 (PK136), -CD11b (M1/70), -F4/80 (BM8), -CD5 (53 – 7.3), -CD9 (MZ3), and -CD138 (281–2) and peritoneal cavity B-1 cells were purifed using biotinylated anti-CD90.2 (30-H12), -CD4 (GK1.5), -CD8a (53 – 6.7), -Gr-1 (RB6–8C5), -NK1.1 (PK136), -CD23 (B3B4), -F4/80 (BM8), - CD5 (53 – 7.3), -CD9 (MZ3), and -CD138 (281–2). The purity of B cells was > 95%, as determined by subsequent flow cytometry analysis.

### B cell cultures

auto-MACS-enriched splenic B-2 or pleural/peritoneal cavity B-1 cells were cultured at 5×10^6^ cells/mL per well or 1×10^6^ cells/mL per well, respectively, in 100 μL of culture media containing RPMI 1640 (Gibco # 21870076), Penicillin-Streptomycin-Glutamine (Gibco # 10378016), FBS (Gibco # 16140071), and β-mercaptoethanol (Gibco # 21985–023) in 96-well round-bottom plates (Falcon # 353077) and stimulated for designated times at 37°C with 5% CO2. Cells were analyzed via flow cytometry with fluorescently labeled antibodies after Fc blocking with anti-CD16/32 (in-house) and Live/Dead staining.

### Macrophage cultures and ex-vivo re-stimulation

auto-MACS-purified lung interstitial macrophages (IMs) or pleural/peritoneal cavity macrophages were cultured at 1×10^6^ cells/mL per well in 100 μL of culture media as described above in 15-mL conical tubes and stimulated for designated times at 37°C with 5% CO_2_. Cells were analyzed via flow cytometry with fluorescently labeled antibodies after Fc blocking with anti-CD16/32 (in-house) and Live/Dead staining. For the detection of TNFα expression, total lung suspension or purified designated macrophages were stimulated with 100 ng/mL of LPS (Sigma # L6511) in the presence of Brefeldin A (Sigma # B6542) for 5 hours at 37°C.

### Single cell RNA-sequencing (scRNA-Seq)

Lung single cell suspension was prepared as above and submitted for FACS-sorting on live cells (FACSAria) using Propidium Iodide (Miltenyi Biotec # 130-093-233) and cells were concentrated at 1000 cell/uL before submitting for 10X single cell RNA Sequencing. Cell viability was ensured to be > 90% prior sequencing submission. For single cell analysis, automatically called cells were further filtered to ensure usage of high-quality droplets with captured cells. RNA barcodes were filtered on Total UMI Count (> 500 UMIs), feature count (> 250 features), and percentage of mitochondrial genes (< 25%). Signac v1.9.0^121^ was used to determine nucleosome signaling and transcription start site enrichment scores. ATAC barcodes were filtered on number of fragments mapping to peak regions (> 3,000 and < 20,000), percentage of fragments mapping to peak regions (> 15%), nucleosome signal scores (< 4) and transcription start site scores (>1).

Seurat v4.3.0^122^ and Signac v1.9.0^121^ used for handling of normalization, identification of variable genes, scaling, principal component analysis, UMAP dimensional reduction, and SNN generation followed by Leiden clustering for RNAseq and ATACseq data respectively. Batch effect correction was performed using Harmony^123^. Clusters were identified using a combination of marker genes and differential expression comparing each cluster to all other cells in the data set. Differential expression analyses were performed using Mann-Whitney U test. fgsea v1.24.0^124^ was used to run gene set enrichment analysis (GSEA) with gene sets obtained from the Molecular Signatures Database^125^. Features were ranked by -log(pvalue) * sign(foldchange).

Percentages of cells by cluster and sample are the percentage of cells in a given cluster compared to the total number of cells per sample. Statistical comparisons shown are from t-test comparing the percentages.

Intercellular signaling analyses are based Domino^126^. UCell^127^ was used to generate transcription factor activation scores by cell using transcription factor target gene sets from the Molecular Signatures Database^125^. ComplexHeatmap v2.14.0^128^ and circlize v0.4.15^129^ were used for visualizations.

### Adaptive transfer experiments

WT or acra7-deficient interstitial macrophages (IMs) were purified using auto-MACS as described above (with purity > 75%) and labeled or not with either eF670 (Thermo Fisher # 65-0840-85) or Cell-trace violet (CTV) (Thermo Fisher # C34571) following the manufacturer’s instructions. After labeling, the cells were washed with PBS, pooled together, and then adoptively transferred into mice intranasally (i.n.) in 20 μL of PBS at the indicated concentrations.

### In vivo treatments

For acetylcholine (ACh) treatment experiments, acetylcholine chloride (Milipore Sigma, # A2661) was diluted in 1X PBS (in-house) to specified concentrations and mixed 1:1 with 1mM of the acetylcholinesterase inhibitor Pyridostigmine Bromide (Milipore Sigma, # P9797–1G). ACh + PB cocktail was then administered in vivo intranasally (i.n.) in 10uL; PBS was used as a control-treated group.

For TNFa blockade experiments, 100mg of anti-TNFa monoclonal blocking antibody^130–132^ (clone XT3.11; BioXcell, # BE0058) was diluted in 1X PBS and administered i.n. in 10uL or intraperitoneally (i.p.) in 50uL. Irrelevant Rat IgG (clone HRPN; BioXcell # BE0088) was used as a control-treated group.

### Quantitative real-time PCR (qRT-PCR) and viral qRT-PCR

For cytokine and chemokine mRNA measurement experiments, 4 small pieces of lung tissue were cut from each lobe and RNA was extracted as per manufacturer instruction (Qiagen, # 69504). All samples were compared to a GAPDH internal control.

For viral qRT-PCR, total lungs were collected and homogenized using gentleMACS ^™^ M Tubes (Miltenyi Biotec, # 130-093-236) in 1mL of PBS, centrifuged, and lung homogenate supernatants were collected, aliquoted and snap-frozen. Viral RNA was purified following manufacturer protocol (Qiagen, # 52904). Presence of influenza viral RNA was detected via quantification of the influenza A M gene using primers: AM-151 (5′ CATGCAATGGCTAAAGACAAGACC-3′) and AM-397 (5′-AAGTGCACCAGCAGAATAACTGAG-3′) and primer/probe AM-245 (6FAM-5′-CTGCAGCGTAGAGCTTTGTCCAAAATG-3′-TAMRA)^133^.

### ELISA assay

Sandwich ELISA assays were conducted following previously established protocols^134^. In brief, ELISA plates were coated with unlabeled anti-isotype antibodies or whole killed influenza A/PR8/34 (200–400 HAU/mL; in-house). To minimize non-specific binding, the plates were blocked with a blocking buffer containing 1% NBCS, 0.1% dried milk powder, and 0.05% Tween 20 in PBS. Serum obtained from tail bleeds or standards were pre-diluted in PBS and then added to the plate in serial dilutions. Antibody detection was achieved using biotinylated anti-isotype antibodies, followed by streptavidin-horseradish peroxidase. The reaction was visualized by adding TMB diluted in 0.05M Citric Acid and H2O2 for 10–15 minutes before stopping with 1N sulfuric acid. Absorbance was measured at 450nm and 595nm, and antibody concentrations were compared to standards. Prior to use in experiments, all reagents were titrated to ensure optimal performance.

### ELISPOT assay

Antibody-secreting cells (ASCs) were quantified using ELISPOT, following established methods^120,134^. Briefly, Multi-Screen HA Filtration plates were coated with unlabeled anti-isotype antibodies or whole killed influenza A/PR8/34 (200–400 HAU/mL; prepared in-house). Following blocking and addition of cell suspensions, antibody secretion was detected using biotinylated anti-isotype antibodies, followed by streptavidin-horseradish peroxidase. The resulting reaction was visualized using a previously described chemical method^134^. Plates were then enumerated using a computer-based system, the AID EliSpot Reader System (Autoimmune Diagnostika).

### Immunofluorescence imaging

For immunofluorescence analysis, B6.Cg-Tg(RP23–268L19-EGFP)2Mik/J (ChAT^BAC^eGFP #007902) mice were first perfused with 1xPBS until clear and then perfused with 4% paraformaldehyde. Tissues were subsequently post-fixed at 4°C for an additional 2 hours. After several washes in 1x PBS, the samples were immersed in 30% sucrose in 1xPBS overnight at 4°C. The following day, the tissues were embedded in Tissue-Tek OCT (Sakura, REF 4583) and frozen on dry ice. Frozen samples were then sectioned at a thickness of 12μm using a cryostat (Leica, CM1850). Sections were initially washed in 1xPBS and then blocked in 10% goat serum (Invitrogen, # 01–6201) containing 0.5% Triton for one hour. Subsequently sections were incubated with primary antibodies overnight at 4°C. The primary antibodies used were Rat anti-B220 (Clone RA3–6B2; 1:200, Biolegend, # 103202) and Rabbit anti-F4/80 (Clone D4C8V; 1:400, Cell Signaling Technology, # 30325T). The next day, slides were washed in 1xPBS three times and then incubated with Alexa Fluor-conjugated, highly cross-adsorbed secondary antibodies along with DAPI (Sigma-Aldrich) for nuclear counterstaining. The goat-derived Alexa Fluor-conjugated secondary antibodies used were anti-rat 647 (1:1000, Invitrogen, A21247) and anti-Rabbit 546 (1:1000, Invitrogen, A10010). Endogenous ChAT-GFP was also examined. Tissue sections were mounted with Aqua-Mount (epredia), and fluorescence images were acquired using a Zeiss 780 LSM confocal microscope.

### Statistical analysis and reproducibility

Statistical analyses were conducted using either GraphPad Prism v8–10 or DESeq2, as specified. Details regarding the statistical methods employed for each analysis are provided within the corresponding figure legends. The precise sample size (n) for each experiment, along with the number of times the experiment was performed and repeated to ensure reproducibility and statistical significance, are also outlined within the figure legends. In summary, most experiments were repeated at least twice and are presented as pooled data or as representatives of two or more repetitions. Blinding procedures were deemed unnecessary for this study, as analyses were conducted using predefined criteria or computationally generated values, with the exception of lung tissue analysis (Extended Data Fig. 7), where blinding was implemented with a single-blinded approach by an expert veterinary pathologist.
